# Effects of Detrital Subsidies on Soft-Sediment Ecosystem Function Are Transient and Source-Dependent

**DOI:** 10.1371/journal.pone.0154790

**Published:** 2016-05-03

**Authors:** Rebecca V. Gladstone-Gallagher, Andrew M. Lohrer, Carolyn J. Lundquist, Conrad A. Pilditch

**Affiliations:** 1 School of Science, University of Waikato, Hamilton, New Zealand; 2 National Institute of Water and Atmospheric Research Ltd. (NIWA), Hamilton, New Zealand; 3 Institute of Marine Science, University of Auckland, Auckland, New Zealand; National Taiwan Ocean University, TAIWAN

## Abstract

Detrital subsidies from marine macrophytes are prevalent in temperate estuaries, and their role in structuring benthic macrofaunal communities is well documented, but the resulting impact on ecosystem function is not understood. We conducted a field experiment to test the effects of detrital decay on soft-sediment primary production, community metabolism and nutrient regeneration (measures of ecosystem function). Twenty four (2 m^2^) plots were established on an intertidal sandflat, to which we added 0 or 220 g DW m^-2^ of detritus from either mangroves (*Avicennia marina)*, seagrass *(Zostera muelleri)*, or kelp (*Ecklonia radiata*) (n = 6 plots per treatment). Then, after 4, 17 and 46 d we measured ecosystem function, macrofaunal community structure and sediment properties. We hypothesized that (1) detrital decay would stimulate benthic primary production either by supplying nutrients to the benthic macrophytes, or by altering the macrofaunal community; and (2) ecosystem responses would depend on the stage and rate of macrophyte decay (a function of source). *Avicennia* detritus decayed the slowest with a half-life (t_50_) of 46 d, while *Zostera* and *Ecklonia* had t_50_ values of 28 and 2.6 d, respectively. However, ecosystem responses were not related to these differences. Instead, we found transient effects (up to 17 d) of *Avicennia* and *Ecklonia* detritus on benthic primary production, where initially (4 d) these detrital sources suppressed primary production, but after 17 d, primary production was stimulated in *Avicennia* plots relative to controls. Other ecosystem function response variables and the macrofaunal community composition were not altered by the addition of detritus, but did vary with time. By sampling ecosystem function temporally, we were able to capture the *in situ* transient effects of detrital subsidies on important benthic ecosystem functions.

## Introduction

In coastal marine systems, detritus (dead, decaying leaf litter) from seagrass, mangroves, salt marsh and macroalgae is transported by the currents, potentially supplying a subsidy to adjacent unvegetated soft-sediment habitats. The role of these detrital subsidies in structuring benthic macrofauna communities in temperate soft-sediments has been well documented and is an important mechanism for creating patchiness and heterogeneity in these recipient habitats (e.g. [1–5]). Furthermore, some studies have indicated that detrital addition increases the biomass of benthic microphytes (e.g. [6–8]), but collectively how these changes influence ecosystem functioning (e.g. benthic primary production, community metabolism, and nutrient regeneration) is not well understood (but see [9]).

Detritus may influence soft sediment ecosystem function via shifts in macrofaunal community composition in response to a new resource, but detritus could also alter nutrient regeneration, and subsequently influence primary production. The degradation of organic matter in soft sediments can increase nutrient regeneration at the sediment-water interface (e.g. [10–14]), fuelling microphytobenthos (MPB) productivity and growth. The observed increases in MPB biomass post-addition of detritus (e.g. [2, 6, 7, 15–17]) may therefore indicate a ‘fertilisation effect’ from the detrital subsidy as a result of nutrient mineralisation during detrital decay [18, 19]. Given that MPB can account for up to 50% of the total estuary autochthonous production [20], this could be an important process maintaining ecosystem productivity. Alternatively, the observed MPB increases may also suggest a removal of grazing pressure through macrofaunal community changes associated with detrital addition (as discussed by [2, 16]). In the field, we explore whether detrital subsidies and the temporal dynamics of decay influence MPB primary production and nutrient regeneration, and whether these associated changes are related to indirect food web effects (i.e. the fertilisation of MPB during detrital decay) or direct macrofaunal community changes in response to detrital subsidies.

Responses of the macrofauna and MPB to detrital addition are dependent on detrital source identity [17, 21], yet questions remain as to how differences in detrital quality (here, defined as the combination of decay rate and C:N content) among macrophyte sources control these responses and the subsequent effects on ecosystem function. The rate of litter decay (an indicator of detrital quality) is likely to influence the magnitude and any corresponding response in the food web. Therefore, any change in ecosystem function in response to detritus could depend on differences in decay rates among detrital sources. For example, in temperate latitudes mangrove leaf litter (e.g. *Avicennia marina*) is refractory and slow to decay (e.g. C:N = 23–47, half-life (t_50_) = 56–157 d; [22, 23]), while macroalgae, on the other hand, is more labile and decays rapidly (e.g. *Macrocystis integrifolia* C:N = 14.3, t_50_ = ~2 weeks; [24]). To explore how differences in the detrital quality among sources may influence soft-sediment ecosystem function, we chose three dominant detrital sources with different decay rates and C:N contents which we added to sediments *in situ*.

Macrophyte detritus decays exponentially, beginning with the rapid leaching of labile materials, which is then followed by the slow degradation of the recalcitrant portion (reviewed by [25]). Despite these important temporal dynamics, previous studies investigating the role of detrital addition on soft sediment ecosystems have mostly considered responses that occur at one or possibly two fixed points in time (most commonly after 2–3 months; e.g. [4, 9, 16, 17, 21, 26, 27]). These studies reveal little about the temporal evolution in ecosystem responses to detrital subsidies associated with the changes that occur during decay. One of the only studies to consider spatio-temporal patterns in macrofaunal community response to detrital additions, revealed significant species specific variations through time [1]. Our experimental design incorporated a temporal element, to explore whether detrital subsidies may have variable effects on benthic ecosystem function.

We added three dominant detrital sources (of different detrital quality) to the sediments on an intertidal sandflat, and then through time measured how these different detrital subsidies influence soft-sediment ecosystem function and benthic macrofaunal community composition. Based on observations that sediment chlorophyll *a* (chl *a*; a measure of MPB biomass) increases with the addition of detritus (e.g. [6, 16]), we expected that detritus would elevate the benthic primary production of MPB, either by releasing nutrients during decay or by altering the macrofaunal community structure. In addition, it was predicted that community metabolism would increase during the aerobic decay of the detritus. We also hypothesised that the magnitude of these ecosystem responses would be dependent on detrital quality, and would vary through time at the different stages of decay. The experiment was designed to increase our understanding of how detrital subsidies contribute to benthic ecosystem function in a field setting.

## Materials and Methods

### Ethics statement

This study complied with all existing legislation governing animal welfare and field-based experiments. Animal ethics approval/permits were not sought as benthic invertebrate fauna sampled in this study are exempt from the Animal Welfare Act 1999. After consultation with the Waikato Regional Council and local iwi representatives, permits were not required for the experiment. The collection of benthic fauna was undertaken with a Ministry of Primary Industries Special Permit (560) Client Number 8770024.

### Experimental treatments and setup

To explore the effects of detrital subsidies on soft-sediment benthic ecosystem function, an experiment was conducted on a mid-intertidal sand flat (tidal elevation ~ +0.5 m above lowest astronomical tide; LINZ data service, Chart NZ 5312) in the Whangapoua Estuary, North Island, New Zealand (S 36° 44' 19.3", E 175° 39' 02.8"). The site was relatively sheltered and not exposed to strong wind wave currents. The sediment at the site consists of organic poor (~ 1% organic content; OC) medium sands, with very little mud (silt/clay particles < 63 μm) content (< 5% by volume). The experiment began in February 2014 (late austral summer) coinciding with peak detrital production and decay [22, 28, 29] and ended in May.

Twenty-four 2 m^2^ (1.4 m × 1.4 m) plots separated by approximately 2 m were established at low tide in a 4 by 6 array. To ensure interspersion, one of the four experimental treatments (three detrital treatments and one control, n = 6 per treatment) was randomly assigned to one plot in each of the rows. Detrital treatments were mangrove (*Avicennia marina* subsp. *australasica*), seagrass (*Zostera muelleri*), and macroalgae (*Ecklonia radiata*) detritus, hereafter referred to as *Avicennia*, *Zostera*, and *Ecklonia* treatments, respectively. At low tide, 220 g m^-2^ of detritus (dry weight, DW) was added to the addition plots, by gently mixing it by hand into the surface sediments (0–5 cm depth) (as in [1, 2, 5, 21]). Control plots were treated in the same manner as detrital plots (i.e. sediments mixed by hand), however no detrital material was added. In addition to the control plots, we measured ecosystem function variables, sediment properties and macrofaunal community structure in ambient undisturbed sediments, to confirm that there were no significant effects caused by the disturbance of finger churning the sediments. The chosen detrital types represent three of the dominant detrital sources present in temperate New Zealand estuaries [30, 31], and include a range of different detrital decay rate and C:N content combinations; from the refractory slow decaying *Avicennia* detritus (C:N = 56, t_50_ = 46 d), to the more labile and rapidly decaying *Ecklonia* detritus (C:N = 18, t_50_ = 3 d), whereas *Zostera* detritus has an intermediate decay rate (C:N = 18, t_50_ = 28 d) (see results).

In order to eliminate treatment effects associated with decay state, the detritus was collected fresh (realistic of what enters the system). Yellow senescent, ready-to-fall leaves were selected from *A*. *marina* trees and live *E*. *radiata* thalli and *Z*. *muelleri* blades were hand-picked. To simulate the natural fragmentation of detritus deposited in the sediments, leaf material was dried at 60°C to constant weight, ground into pieces ~ 2 mm in dia. and stored (< 2 weeks) before addition to the plots. The drying process is thought to be similar to that experienced by washed up detrital material during a summer afternoon low tide (e.g. [17]), and enabled us to standardise the amount and surface area of detritus added to each plot.

At 4, 17 and 46 d post-detrital addition, we measured benthic solute fluxes across the sediment-water interface, as well as macrofaunal community structure and sediment properties in each of the 24 plots. A different (randomly selected) quarter (0.5 m^2^) of each square plot was sampled on each date. Sampling times were chosen to encompass sedimentary and macrofaunal responses associated with the initial leaching and decay that litter experiences during decomposition [22, 23], as well as the possible longer-term effects on macrofauna identified in previous studies (e.g. [21, 26]). In order to determine the variability in ambient light and temperature levels between sampling dates, four HOBO data loggers (5 min. sampling interval) were placed within the study site during solute flux measurements. To determine source-specific decay rates for our study location, litterbags were positioned on the sediment surface (16 cm × 16 cm, 2mm mesh; [22, 28]) with a known initial DW of detritus. Litterbags were then retrieved at 4, 17, and 46 d post-addition (n = 4 bags per detrital type, per retrieval date). To eliminate decay effects associated with differences in the leaf surface area, and therefore obtain a relative decay rate between the detrital sources, we shredded the detritus for the litterbags to ensure that all types had a similar surface area to seagrass blades.

### Field measurements

During a midday high tide, *in situ* benthic chambers were used to measure fluxes of dissolved oxygen and inorganic nutrients across the sediment-water interface (as in [12, 32]). In each plot, two circular chambers (one transparent ‘light’, and one blacked out ‘dark’) were placed side-by-side on an incoming tide incubating the sediment and overlying water (chamber sediment surface area = 0.016 m^2^, water vol. = 0.85 L). Each chamber had a sampling port and an inlet port that allowed ambient water to enter the chamber during sample extraction. After flushing with ambient seawater, the chambers were incubated for approximately 4 h (2 h before and after high tide) with water samples collected at the start and end of the incubation period. For each sample, the first 20 ml of water withdrawn from the chamber was discarded (i.e. water contained in the 1.5 m of sample tubing) before a further 60 ml sample was collected for analysis. To account for water column processes in our chamber flux calculations, three pairs of light and dark 1.5 L bottles were filled with ambient seawater, incubated just above the seabed, and sampled at the same time as the benthic chambers. Immediately following water sample collection, dissolved oxygen concentration was measured using an optical DO probe (PreSens Fibox 3 PSt3), then the sample filtered through a 24 mm Whatman GF/C filter, and immediately frozen awaiting analysis of dissolved inorganic nutrients.

After completion of the chamber incubations, one core (13 cm dia. × 15 cm depth) was collected from under the dark chamber in each plot, and the material retained on a 500 μm mesh sieve preserved in 70% Isopropyl alcohol for macrofaunal community analysis. Surface sediment properties (chl *a*, OC, and grain size—GS) were measured in each plot by taking three pooled sediment cores (3 cm dia. × 2 cm depth). Sediment samples were transported back to the laboratory on ice and then frozen prior to analysis. To reduce the disturbance created by sampling, core holes were infilled with defaunated sand (as in [32]).

### Laboratory Analyses

Filtered water samples were analysed for dissolved inorganic nutrient species (NH_4_^+^, NO_3_^-^, NO_2_^-^, PO_4_^3-^) on a LACHAT Quickchem 8500 series 2 Flow Injection Analyser (FIA). Sediment chl *a* and phaeophytin (Phaeo) pigments were extracted using 90% buffered acetone, and concentrations (μg g^-1^) were determined on a Turner 10-AU fluorometer, before and after acidification [33]. Sediment OC was determined by weight loss on ignition, after drying at 60°C to constant weight and then subsequent combustion at 550°C for 4 h. Sediment GS was measured using a Malvern Mastersizer 2000 (Particle size range: 0.05–2000 μm), following organic matter digestion in 10% hydrogen peroxide. Macrofauna were separated from sediment and shell hash after staining with Rose Bengal stain, and then identified to the lowest feasible taxonomic level (usually species). To quantify the amount of detritus remaining in plots, macrofaunal core samples (with the fauna removed) were elutriated in a sugar solution to separate the less dense detrital material from heavier shell hash and sediment [34]. Elutriated material was dried to constant weight at 60°C and then weighed. Litterbag samples were washed, dried at 60°C to constant weight and then weighed, to determine percentage weight loss through time. In addition, the initial C and N content in each detrital source was measured (n = 3) using an Elementar–vario EL cube analyser.

### Flux calculations and data analysis

Fluxes of dissolved oxygen and inorganic nutrients across the sediment-water interface were calculated by subtracting the initial from the final concentration, and standardising this difference by incubation time, chamber water volume, and the enclosed sediment surface area. Chamber fluxes were also corrected for water column processes (mostly < 5% of the measured chamber flux). These fluxes were used to derive the following measures of ecosystem function: net primary production (NPP; light chamber O_2_ flux), sediment oxygen consumption (SOC), which is used as a proxy for benthic community metabolism/respiration in the absence of MPB photosynthesis (dark chamber O_2_ flux), and gross primary production (GPP; light minus dark chamber O_2_ flux). Normalising GPP by sediment chl *a* content accounts for variation in MPB biomass providing an estimate of photosynthetic efficiency (GPP_chl a_). Concentrations of NO_2_^-^, NO_3_^-^, and PO_4_^3-^ were below or near detection limits (0.004 mg L^-1^) resulting in uncertainty and variability in flux calculations, therefore these nutrient species were not considered further. NH_4_^+^ fluxes in light and dark chambers were considered a proxy for inorganic nutrient regeneration in this study, as NH_4_^+^ is the first nitrogenous product of organic matter remineralisation and is linked to MPB production in New Zealand estuaries (e.g. [35, 36]). Preliminary analysis of NH_4_^+^ fluxes showed no significant difference between the light and dark chambers (PERMANOVA, *p* = 0.3) on any sampling dates, so were averaged for each light-dark chamber pair prior to statistical analysis.

t-tests were used to confirm that there was no procedural effect by comparing univariate response variables (sediment properties, solute fluxes, macrofauna abundance/richness) between ambient and control plots on d 4. t-tests were performed in the STATISTICA software package (Statsoft Inc.) on untransformed data after checking that the data met assumptions of independence, normality, and homogeneity of variance. In addition, a multivariate one-factor permutational analysis of variances (PERMANOVA) based on a Bray-Curtis similarity matrix was used to compare the macrofaunal community structure between ambient sediments and control plots.

We used a repeated measures PERMANOVA to determine treatment effects through time on each univariate response variable (OC, chl *a*, phaeo, median GS, mud content, detritus remaining, macrofauna abundance and taxa richness, NH_4_^+^, SOC, NPP, GPP, GPP_chl a_; using Euclidean distance matrices), as well as the multivariate macrofauna data (Bray-Curtis similarity), and multivariate sediment properties (OC, chl *a*, phaeo, median GS, mud content; Euclidean distance). The analysis had treatment (4 levels) and time (3 levels) as fixed factors, and plot (6 levels) as a random factor nested within treatment. As our hypotheses were based upon an anticipated temporal succession in treatment effects, time was considered a fixed (treatment) factor [37]. Main effects (treatment and time) were not considered if the time × treatment interaction was significant, instead post-hoc pair-wise tests were undertaken to identify differences between treatment effects for each sampling date. In the absence of a time × treatment interaction, pair-wise tests determined differences between treatments and sampling dates. Non-metric Multidimensional Scaling analysis (nMDS) was used to visualise patterns in multivariate macrofaunal community species data among treatments and sampling dates, and SIMPER analysis used to determine which species were contributing to community differences. Raw, untransformed macrofauna species data were used in PERMANOVA and nMDS analyses, because abundances were spread relatively evenly across taxa, making transformations unnecessary. Univariate response variables were also left untransformed. PERMANOVA, nMDS and SIMPER analyses were all performed in the PRIMER 7 statistical software program [37, 38].

Single exponential decay models (*X*_*(t)*_ *= e*^*-kt*^; [25]) were used to estimate decay rates of the detritus using untransformed data collected at 4, 17 and 46 d. In the model, *X*_*(t)*_ = the proportion of detritus remaining in the litterbags after time *t* (days), and *k* = detrital decay constant (d^-1^). In using the litterbag method, decay represents not only decomposition, but the potential loss of litter pieces that are smaller than the litterbag mesh (< 2 mm). t_50_ (i.e. time in days it takes for the detritus to decay to half its original weight) was then calculated as: *t*_*50*_ *= k*^*-1*^ *× ln2*, along with the 95% confidence intervals of the decay curves. Decay models were fitted using STATISTICA (Statsoft Inc.). All raw data used in analyses can be found in the supporting information (S1 Table, S2 Table, S3 Table).

## Results

We found no procedural effects (of hand mixing the sediments) on the sediment properties (Table 1) and ecosystem function variables (GPP, NPP, SOC, GPP_chl a_ and NH_4_^+^ flux) in t-tests comparing control and ambient sediments after 4 d (t-tests *p* > 0.3). Sediment mixing had no effect on the macrofaunal community structure (PERMANOVA df = 1, pseudo-F = 0.6, *p* = 0.7), total abundance or taxa richness (t-tests *p* > 0.4). Therefore, results measured from ambient plots were excluded from all further analyses.

**Table 1:** Sediment properties and macrofaunal community variables. Variables are reported as a function of detritus treatment (control, *Avicennia*, *Zostera*, *Ecklonia*) and time (4, 17, 46 d post-detrital addition). Day 4 ambient data were included to test for procedural effects (see text) and data represent the mean ±1 SE (n = 6 (4 for ambient plots)).

Day	Variable	Ambient	Control	*Avicennia*	*Zostera*	*Ecklonia*
4	OC (%)	1.08 ± 0.07	1.11 ± 0.03	1.48 ± 0.06	1.35 ± 0.06	1.26 ± 0.03
	Chl *a* (μg g^-1^)	7.5 ± 1.0	7.2 ± 0.4	6.4 ± 0.3	7.0 ± 0.7	6.5 ± 1.1
	Phaeo (μg g^-1^)	3.6 ± 1.2	3.9 ± 0.6	6.4 ± 0.8	5.6 ± 0.6	8.7 ± 1.1
	Mud content (%)	2.5 ± 1.0	3.1 ± 0.7	3.0 ± 0.6	3.0 ± 0.2	2.7 ± 0.4
	Median GS (μm)	274 ± 7	265 ± 5	266 ± 5	261 ± 3	263 ± 4
	Amount of detritus (g DW core^-1^)	0.35 ± 0.17	0.49 ± 0.13	0.84 ± 0.17	1.42 ± 0.63	0.57 ± 0.10
	Macrofauna total abundance (core^-1^)	206 ± 57	175 ± 24	218 ± 39	177 ± 27	218 ± 25
	Macrofauna taxa richness (core^-1^)	20.8 ± 1.5	18.8 ± 1.6	19.8 ± 1.7	19.5 ± 0.9	20.0 ± 0.8
17	OC (%)		1.18 ± 0.15	1.24 ± 0.05	1.38 ± 0.10	1.19 ± 0.08
	Chl *a* (μg g^-1^)		7.5 ± 1.10	6.3 ± 0.4	9.1 ± 1.3	5.9 ± 0.5
	Phaeo (μg g^-1^)		6.9 ± 1.3	7.1 ± 1.1	5.6 ± 1.1	6.5 ± 1.5
	Mud content (%)		3.1 ± 0.2	3.3 ± 0.6	3.5 ± 0.4	3.7 ± 0.3
	Median GS (μm)		265 ± 3	263 ± 4	255 ± 3	264 ± 4
	Amount of detritus (g DW core^-1^)		0.35 ± 0.09	1.04 ± 0.50	0.94 ± 0.20	0.56 ± 0.15
	Macrofauna total abundance (core^-1^)		226 ± 24	239 ± 17	269 ± 19	291 ± 24
	Macrofauna taxa richness (core^-1^)		25.2 ± 2.2	22.0 ± 0.8	22.7 ± 1.5	25.0 ± 1.2
46	OC (%)		1.23 ± 0.09	1.21 ± 0.03	1.34 ± 0.02	1.16 ± 0.10
	Chl *a* (μg g^-1^)		7.9 ± 0.4	7.51 ± 1.2	8.49 ± 1.1	7.71 ± 1.7
	Phaeo (μg g^-1^)		4.4 ± 0.8	4.4 ± 0.5	4.5 ± 0.8	4.0 ± 0.7
	Mud content (%)		2.4 ± 0.5	2.7 ± 0.2	3.0 ± 0.5	2.9 ± 0.5
	Median GS (μm)		265 ± 3	275 ± 5	264 ± 6	266 ± 4
	Amount of detritus (g DW core^-1^)		0.61 ± 0.27	0.38 ± 0.11	1.00 ± 0.41	0.42 ± 0.08
	Macrofauna total abundance (core^-1^)		183 ± 21	200 ± 19	203 ± 31	202 ± 15
	Macrofauna taxa richness (core^-1^)		17.3 ± 0.5	20.2 ± 1.0	21.5 ± 1.4	21.3 ± 0.8

OC = total organic content of sediment; Chl *a* = sediment chlorophyll *a* pigment content; Phaeo = sediment phaeophytin pigment content; GS = grain size; Mud = silt/clay (particles < 63 μm); DW = dry weight

### Sediment variables

Four days post-detrital addition, sediment OC was elevated by 11–33% in treatment plots relative to the controls (Table 1). A similar pattern was also seen in the amount of detritus recovered (by sugar elutriation), where addition plots were elevated by 14–65% compared to controls. These increases in OC and detritus recovered however were only statistically significant for *Zostera*, which remained elevated throughout the experiment (Table 2).

**Table 2:** Repeated measures PERMANOVA results for sediment properties and macrofauna community variables. PERMANOVA tests were performed on univariate measures of sediment properties, macrofaunal abundance, and taxa richness (Euclidean Distance), and multivariate macrofaunal community structure (Bray Curtis similarity), as a function of time (4, 17, 46 d post-addition) and treatment (C = control, A = *Avicennia*, E = *Ecklonia*, Z = *Zostera*). Significant effects (*p* < 0.05) are indicated in bold. In the instance of time × treatment interactions, *p* values are not given for main effects, and PERMANOVA post-hoc pair-wise tests show treatment effects on each sampling date, separately.

Variable	Source	df	MS	Pseudo-F	*p*(perm)	Post-hoc pair-wise tests
OC	Time × Treatment	6	0.05	2.13	0.0676	
	Time	2	0.03	1.16	0.3233	
	Treatment	3	0.14	3.48	**0.0387**	C = A, C = E, C<Z, A = E, A = Z, E<Z
	Plot(treatment)	20	0.04	1.67	0.0784	
	Residual	40	0.02			
Chl *a*	Time × Treatment	6	2.56	0.83	0.5652	
	Time	2	7.75	2.50	0.0924	
	Treatment	3	8.92	5.77	**0.0041**	C = A, C = E, C = Z, A = E, A<Z, E<Z
	Plot(treatment)	20	1.54	0.50	0.9617	
	Residual	40	3.11			
Phaeo	Time × Treatment	6	10.05	2.37	**0.0433**	4 d: C<A, C<E, C = Z, A = E, A = Z, E>Z;
	Time	2	32.78	7.74		17 and 46 d: ns
	Treatment	3	7.10	1.38		
	Plot(treatment)	20	5.14	1.21	0.2896	
	Residual	40	4.23			
Mud content	Time × Treatment	6	0.41	0.55	0.7725	
	Time	2	2.55	3.47	**0.0418**	4 d = 17 d, 4 d = 46 d, 17 d>46 d
	Treatment	3	0.34	0.23	0.8913	
	Plot(treatment)	20	1.46	1.99	**0.0319**	
	Residual	40	0.73			
Median GS	Time × Treatment	6	56.54	1.20	0.3310	
	Time	2	214.30	4.56	**0.0152**	4 d = 17 d, 4 d = 46 d, 17 d<46 d
	Treatment	3	184.66	1.14	0.3610	
	Plot(treatment)	20	162.21	3.46	**0.0005**	
	Residual	40	46.95			
Amount of detritus	Time × Treatment	6	0.31	0.70	0.6725	
	Time	2	0.32	0.71	0.5234	
	Treatment	3	1.56	3.98	**0.0181**	C = A, C = E, C<Z, A = E, A = Z, E<Z
	Plot(treatment)	20	0.39	0.87	0.6202	
	Residual	40	0.45			
Macrofauna total	Time × Treatment	6	1949.90	0.63	0.7006	
abundance	Time	2	28342.00	9.23	**0.0005**	4 d<17 d, 4 d = 46 d, 17 d>46 d
	Treatment	3	5478.10	1.87	0.1681	
	Plot(treatment)	20	2929.40	0.95	0.5265	
	Residual	40	3071.80			
Macrofauna taxa	Time × Treatment	6	15.08	2.20	0.0621	
richness	Time	2	128.43	18.70	**0.0001**	4 d<17 d, 4 d = 46 d, 17 d>46 d
	Treatment	3	9.20	0.75	0.5339	
	Plot(treatment)	20	12.25	1.78	0.0590	
	Residual	40	6.87			
Macrofaunal community	Time × Treatment	6	366.81	0.81	0.7831	
(Multivariate)	Time	2	3614.10	8.02	**0.0001**	4 d≠17 d, 4 d≠46 d, 17 d≠46 d
	Treatment	3	494.18	0.80	0.7174	
	Plot(treatment)	20	620.46	1.38	**0.0122**	
	Residual	40	450.53			

OC = total organic content of sediment; Chl *a* = sediment chlorophyll *a* pigment content; Phaeo = sediment phaeophytin pigment content; GS = grain size; Mud = silt/clay (particles < 63 μm)

Other sediment properties were mostly unaffected by the detrital addition, except for chl *a* and phaeo. Chl *a* was consistently higher in *Zostera* plots compared to *Avicennia* and *Ecklonia* plots, but none of the detritus treatments differed from controls. Phaeo was higher in *Avicennia* and *Ecklonia* plots relative to controls after 4 d, but no treatment effects were observed 17 and 46 d post-addition. Mud content and median GS differed between sampling dates (Tables 1 and 2). A multivariate PERMANOVA analysing treatment and time effects on all sediment properties combined revealed no treatment effects (df = 3, pseudo-F = 1.18, *p* = 0.3), but significant time effects were found (df = 2, pseudo-F = 4.68, *p* = 0.01), and post-hoc pair-wise tests revealed that multivariate sediment properties at 46 d were significantly different to those at 4 and 17 d (*p* < 0.05).

### Detrital decomposition

**Fig 1:**
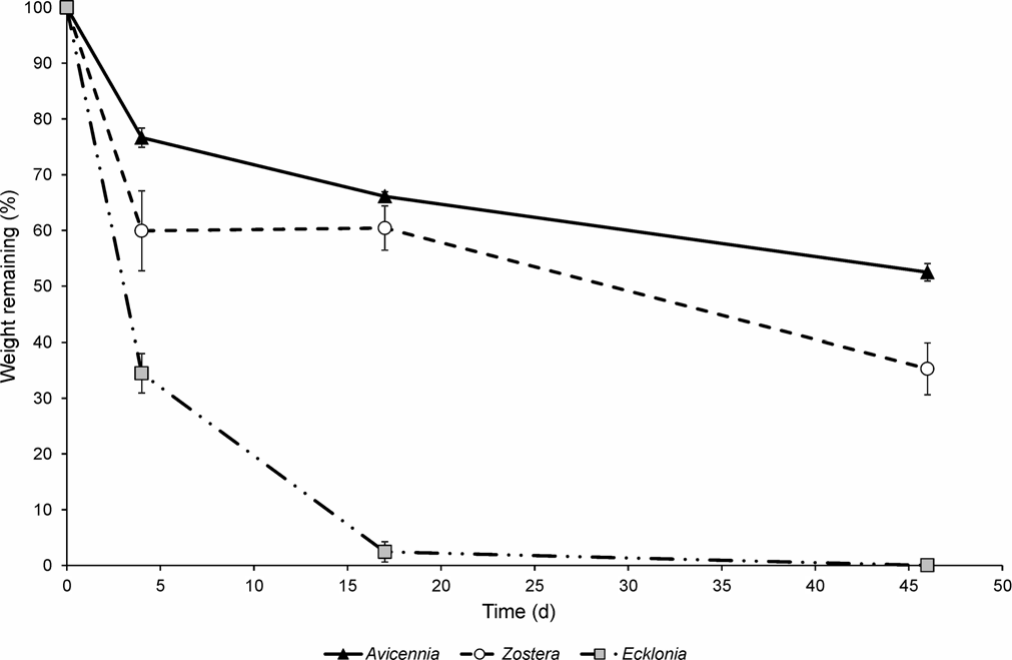
Decay rates of *Avicennia*, *Zostera* and *Ecklonia* detritus. Data represent the mean percentage (±1 SE; n = 4) of initial dry weight (DW) remaining in litterbags as a function of time.

Initial litter C:N ratios (±1 SE, n = 3) were 55.9 (±0.3) for *Avicennia* (N = 0.82%), 18.49 (±0.06) for *Zostera* (N = 1.49%), and 18.39 (±0.06) for *Ecklonia* (N = 1.83%). Leaf litterbag results confirmed distinct differences in detrital decay rates among *Avicennia*, *Zostera*, and *Ecklonia* detritus. After 46 d, *Avicennia* lost 48% of its weight, *Zostera* litter 65%, and *Ecklonia* decayed the fastest with no litter left at the end of the experiment (Fig 1). These differences in weight lost were reflected in t_50_ values (95% CI), which were 46 d (41–53 d), 28 d (23–37 d), and 2.6 d (2.5–2.8 d) for *Avicennia*, *Zostera*, and *Ecklonia* detritus, respectively.

### Macrofaunal community

We collected 52 different macrofaunal species/taxa, with a total of 16,425 individuals across the 24 plots on three sampling occasions. The dominant group were the polychaetes, making up 54% of the total abundance comprising 20 species. Of the remaining groups, bivalves contributed 23% to the total abundance (6 species), amphipods 8% (8 species), gastropods 4% (8 species), with the remainder (~ 10%) in the classes anthozoa, crustacea (orders not including amphipoda), rhabditophora, polyplacophora, clitellata and nemertea, all of which had just 1–2 species each.

**Fig 2:**
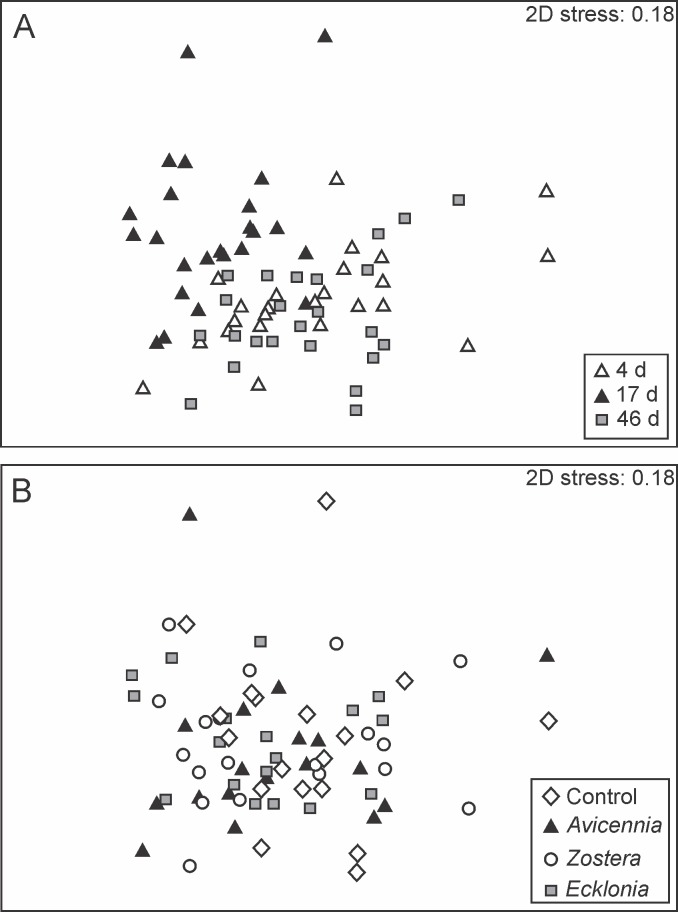
nMDS ordination of untransformed macrofaunal community data. Ordinations (based on Bray-Curtis similarity) show species distributions as a function of **(A)** time: 4, 17 and 46 d post-detrital addition (n = 24) and **(B)** detrital treatments: control, *Avicennia*, *Zostera*, and *Ecklonia* (n = 18). Each data point represents the macrofaunal community in one core sample.

Multivariate macrofaunal community structure, and univariate abundance and richness changed through time (Tables 1 and 2, Fig 2A). Pair-wise tests revealed that univariate measures of abundance and taxa richness were higher on d 17 compared to d 4 and 46, whereas multivariate community structure differed among all three sampling dates. SIMPER analysis showed that the same species (the polychaetes *Prionospio aucklandica* and *Aonides trifida*, bivalves *Austrovenus stutchburyi* and *Lasaea parengaensis*, and amphipod *Paracalliope novizealandiae*) were responsible for 50% of the cumulative dissimilarity between sampling dates, indicating that temporal differences in community structure were likely driven by changes in the relative abundances of these species. No significant effects of detrital addition on univariate or multivariate measures of macrofaunal community structure were detected (Table 2, Fig 2B).

### Measures of ecosystem function

**Fig 3:**
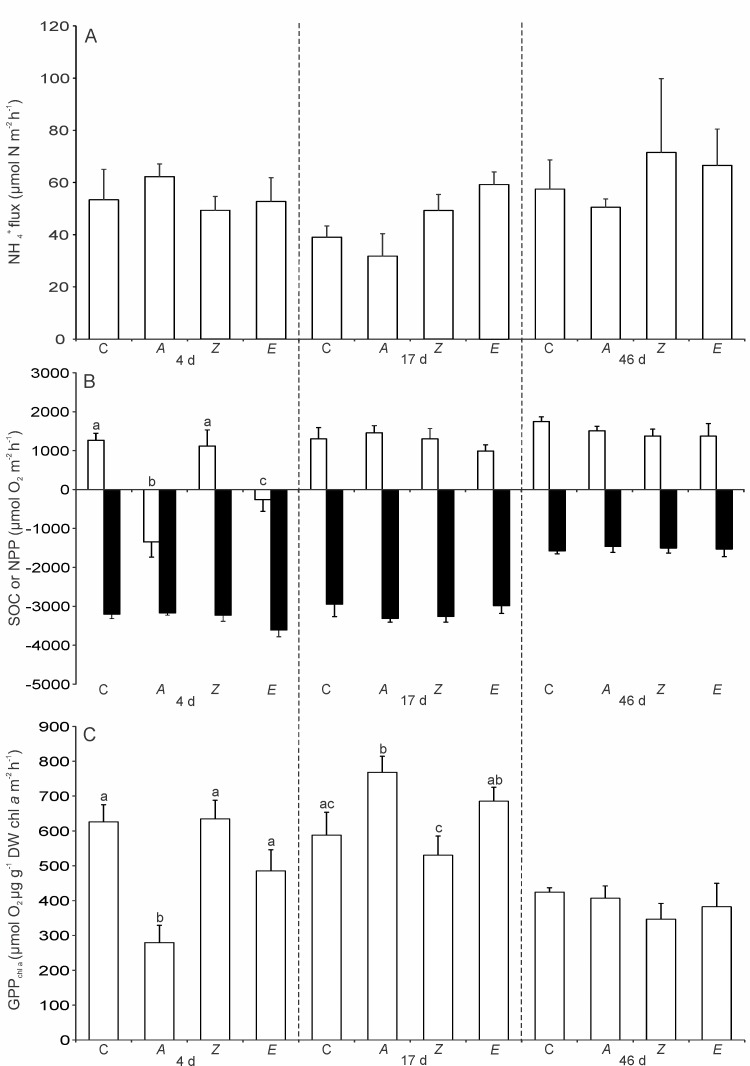
Solute fluxes in control and detrital treatments at 4, 17, and 46 d post-addition. **(A)** NH_4_^+^ flux (light and dark chamber fluxes pooled); **(B)** Net primary production (NPP; white bars light chambers) and sediment oxygen consumption (SOC; black bars dark chambers); and **(C)** Gross primary production normalised for chlorophyll *a* biomass (GPP_chl a_), as a function of treatment (C = Control, *A* = *Avicennia*, *Z* = *Zostera*, *E* = *Ecklonia*) and time (4, 17, and 46 d post-addition). Data represent the mean +1 SE (n = 6). PERMANOVA pair-wise test results (within a sampling date) for significant time × treatment interaction are shown as letters above bars, where bars sharing the same letter are not significantly different (*p* < 0.05).

NH_4_^+^ flux and SOC were unaffected by the addition of detritus throughout the experiment, but both showed significant temporal variability (Table 3, Fig 3A and 3B). The NH_4_^+^ flux was higher (19–26%) on d 4 and 46 compared to d 17. The SOC measured at 4 and 17 d post-detrital addition was double that measured on d 46. Light levels at the sediment-water interface and salinity also varied across the sampling dates (Table 4).

**Table 3:** Summary of repeated measures PERMANOVA results on univariate measures of ecosystem function. PERMANOVA tests (Euclidean distance) were performed on ecosystem function variables, as a function of time (4, 17, 46 d post-addition) and treatment (C = control, A = *Avicennia*, E = *Ecklonia*, Z = *Zostera*). Significant effects (*p* < 0.05) are indicated in bold. In the instance of time × treatment interactions, *p* values are not given for main effects, and PERMANOVA post-hoc pair-wise tests show treatment effects on each sampling date, separately.

Ecosystem function variable	Source	df	MS	Pseudo-F	*p*(perm)	Post-hoc pair-wise tests
NH_4_^+^	Time × Treatment	6	3542	1.21	0.2883	
	Time	2	7914	2.71	**0.0362**	4 d>17 d, 4 d = 46 d, 17 d<46 d
	Treatment	3	2175	0.76	0.6051	
	Plot(treatment)	20	2867	0.98	0.5024	
	Residual	40	2923			
SOC	Time × Treatment	6	211230	1.60	0.1711	
	Time	2	23157000	175.84	**0.0001**	4 d = 17 d, 4 d>46 d, 17 d>46 d
	Treatment	3	53999	0.37	0.7813	
	Plot(treatment)	20	147280	1.12	0.3716	
	Residual	40	131690			
NPP	Time × Treatment	6	3106900	9.33	**0.0001**	4 d: C>A, C>E, C = Z, A<E, A<Z, E<Z;
	Time	2	11620000	34.88		17 and 46 d: ns
	Treatment	3	3376000	9.52		
	Plot(treatment)	20	354700	1.06	0.4158	
	Residual	40	333140			
GPP	Time × Treatment	6	3512100	6.94	**0.0001**	4 d: C>A, C>E, C = Z, A<E, A<Z, E = Z;
	Time	2	2767900	5.64		17 d: C = A, C = E, C = Z, A>E, A = Z, E = Z;
	Treatment	3	490980	0.97		46 d: C<A, C = E, C = Z, A = E, A = Z, E = Z
	Plot(treatment)	20	490980	0.97	0.5094	
	Residual	40	505960			
GPP_chl a_	Time × Treatment	6	113300	7.85	**0.0001**	4 d: C>A, C = E, C = Z, A<E, A<Z, E = Z;
	Time	2	11896	1.28		17 d: C<A, C = E, C = Z, A = E, A>Z, E>Z; 46d: ns
	Treatment	3	9264	0.64		
	Plot(treatment)	20	9264	0.64	0.8593	
	Residual	40	14437			

NH_4_^+^ = ammonium flux; SOC = sediment oxygen consumption; NPP = net primary production; GPP = gross primary production; GPP_chl a_ = GPP normalised for chlorophyll *a* biomass

**Table 4:** Light, temperature, and salinity at the sediment-water interface. For light and temperature, the mean (±1 SE; n = 4 loggers) for each incubation period is presented, and for salinity, the results of a single measurement are shown.

Day	Light (Lux)	Temperature (°C)	Salinity
4	12493 ± 3828	22.2 ± 0.1	25.2
17	22282 ± 12130	20.1 ± 0.1	30.7
46	5573 ± 1138	20.1 ± 0.1	24.3

Ecosystem function variables related to primary production (NPP, GPP, GPP_chl a_) showed significant time × treatment interactions (Table 3), indicating that detrital treatment effects varied among the sampling dates. PERMANOVA pair-wise comparisons revealed that 4 d after the addition, NPP was lower in *Avicennia* and *Ecklonia* treatments compared to that measured in control and *Zostera* plots (Table 3; Fig 3B). In *Avicennia* and *Ecklonia* treatments, there was a drawdown of oxygen into the sediments (a negative flux of ~ -260 to -1350 μmol O_2_ m^-2^ h^-1^) while in the control and *Zostera* treatments there was an efflux of oxygen out of the sediments and into the water column (a positive flux ~ 1200 μmol O_2_ m^-2^ h^-1^). However, these treatment effects on NPP were not found on subsequent sampling dates. Like NPP, GPP was reduced in *Avicennia* (by 59%) and *Ecklonia* (by 23%) plots compared to control plots, but only on d 4. GPP_chl a_ was reduced by similar amounts on d 4 in *Avicennia* and *Ecklonia* (marginally significant at *p* = 0.09) plots, but interestingly after 17 d *Avicennia* plots had higher GPP_chl a_ (by 23%) compared to control plots. After 46 d, there was no detrital treatment effects on GPP_chl a_ (Table 3; Fig 3C).

## Discussion

Previous studies have highlighted the role that macrophyte detrital subsidies play in structuring benthic macrofaunal communities and influencing MPB biomass on temperate intertidal flats (e.g. [1, 16, 21, 26, 27]). This study, however, is the first to measure the temporal succession of *in situ* benthic primary production, community metabolism, and nutrient regeneration following the addition of detritus to the sediments. Four days after the addition, sediment OC was raised in detrital treatment plots relative to controls (by 11–33%), though this was only significant for *Zostera*, which remained raised throughout the experiment. Ecosystem responses to detrital additions however were not as predicted from their differences in C:N ratios and decay rates. We expected that the responses among detrital sources would vary through time due to differences in detrital quality, and that initially the fastest decaying, most labile detrital source (*Ecklonia*) would show the greatest response in ecosystem function, with the slowest decaying (*Avicennia*) having the least response. Instead, *Avicennia* and *Ecklonia* detritus (t_50_ = 46 and 2.6 d, respectively) both influenced short-term primary production of the sediments, with no effects of the addition of *Zostera* detritus (t_50_ = 28 d), and these effects changed as the experiment progressed. Nutrient regeneration, community metabolism, and the macrofaunal community showed no response to the addition of detritus, but were instead dominated by temporal changes.

Our measures of community metabolism (SOC) and nutrient regeneration (NH_4_^+^ flux) varied through time and were unaffected by detrital enrichment (or the interaction of these two factors). Macrofauna are known to regulate ecosystem functions, such as SOC and NH_4_^+^ fluxes [14, 32, 39–41], and the subtle shifts in the relative abundances of a few species among the sampling dates (e.g. high abundances at 17 d) may be responsible for the temporal changes in NH_4_^+^ flux. Furthermore, correlations between sediment properties and ecosystem functions, such as SOC, have been found previously (e.g. [41]), and in our study, the temporal differences in several sediment properties could explain the differences we found in SOC (i.e. both multivariate sediment properties and SOC changed on 46 d).

Unlike SOC and NH_4_^+^, ecosystem functions associated with benthic primary production (NPP, GPP, GPP_chl a_) showed significant time × treatment interactions, revealing that detrital enrichment effects changed and evolved through time. It is common for soft-sediment communities to show temporal variation (e.g. [42, 43]), and it has been suggested that heterogeneity in soft-sediment ecosystems contributes to ecosystem stability and resilience [44–46]. Our results have found that detritus creates transient responses in function, therefore potentially contributing to the heterogeneous nature of intertidal sandflat ecosystems. Here, we demonstrate that sampling at one point in time gives us only a snap-shot of benthic ecological function, while omitting important transient processes that evolve over varying time scales in response to detrital decay processes. Our detrital decay curves show that the initial rapid leaching stage [25] occurred in the first 4 days of decay for all sources, which was then followed by the slow decay of the recalcitrant components of the leaf. Detritus-induced changes to benthic primary production are likely associated with the time scales of decay, which may explain the changes in primary production through time that we detected (e.g. the initial suppression of primary production at 4 d).

Source dependent detrital effects were not related to differences in detrital decay rate, and instead the fastest and slowest decaying sources (*Ecklonia* and *Avicennia*) were the sources to have effects on sediment primary production. This suggests that detrital responses may be controlled by the chemical composition and palatability of the detrital source, rather than the decay rate. The initial suppression (4 d) of NPP, GPP and GPP_chl a_ in *Avicennia* and *Ecklonia* was unexpected, given our prediction that detrital subsidies could ‘fertilise’ and stimulate MPB primary production. The absence of treatment effects on SOC in the dark chambers mean that treatment differences in GPP and GPP_chl a_ are associated with changes in the light chambers (NPP), where photosynthesis by MPB occurs. Both mangrove and kelp detritus contain secondary chemical compounds (deterrents for consumers), such as tannins, that leach during decomposition [47]. This leaching of plant compounds may be responsible for the short-term suppression in GPP and GPP_chl a_, either in a photo-inhibitory manner as the brown colour of leached compounds may inhibit light reaching MPB (we observed the brown colour in our plots at 4 d), or through toxic effects on MPB. Secondary compounds in mangrove leaves, such as tannins, have been shown to have negative effects on soft sediment meiofauna [48], and it is possible that they have similar negative effects on MPB, though this requires further investigation. After 17 d, *Avicennia* detritus significantly increased GPP_chl a_ (but not GPP), possibly due to a ‘fertilisation effect’ as the detritus slowly decays [18, 19]. However, this increase in GPP_chl a_ was not associated with any changes in macrofaunal community, and therefore we hypothesise that the response was instead microbial.

We expected to see shifts in macrofaunal community structure with detrital enrichment that have been found previously (e.g. [3, 6, 27]), but these responses were absent at our site. Site-dependent macrofaunal responses have been found by others (e.g. [7, 17]), and our results confirm that macrofaunal responses to detrital enrichment must be context-specific, and are perhaps regulated by the resident macrofaunal community or sediment type. Significant shifts in macrofaunal abundances and species compositions have been noted in sites with muddy sediments (e.g. [1, 16, 17, 21, 27]). We note that our study site had relatively sandy sediments, which generally have low background organic content compared to mud [41]. Increased organic loading in mud may induce greater microbial and macrofaunal responses associated with reaching a threshold of organic enrichment and anoxia, that may not occur in organic poor sands. Additionally, specific species are responsible for detrital induced faunal community changes, and these have included deposit-, scavenger- and suspension-feeding species from families Capitellidae, Cirratulidae, Orbiniidae, Nereididae, and Oligochaeta, as well as the sabellid polychaete, *Euchone variabilis*, and the bivalve, *Macomona deltoidalis* [1, 3, 7, 17, 27]. While some of these taxa (i.e. species from the same family) were present at our site in low abundances (e.g. Capitellidae, Orbiniidae, Nereididae, Oligochaeta, bivalve *Macomona liliana*), others were absent (Sabellidae, Cirratulidae), and perhaps our resident macrofaunal community was not supported by a detrital based food web. Studies across multiple sites have demonstrated that macrofaunal species which respond to detritus at some sites do not always respond at other sites [17].

The lack of response by the macrofaunal community to the detrital additions may be a function of the amount added. However, the amount (220 g DW m^-2^) and the form (shredded) of the added detritus is comparable to other studies that found significant macrofaunal responses (e.g. [1, 3, 16]). It is possible that the more productive sandy communities [41] are perhaps less reliant on detritus as a primary food source than muddy communities. The productive MPB offer a palatable source of lipids and proteins for benthic consumers, whereas macrophyte detritus contains complex structural carbohydrates that must go through a microbial pathway before they can be effectively ingested. Therefore, in many estuaries the benthic food web is thought to be supported by MPB, which is more efficiently assimilated and nutritious (reviewed by [49]).

We show that on a small spatial scale (2 m^2^), soft-sediment ecosystem responses to detrital addition are short-term, temporally variable, and macrophyte source-dependent. The detrital effects we saw in the benthic primary production suggest that detrital subsidies are likely to contribute to the transient and heterogeneous nature of temperate sandflats by altering important ecosystem functions. Further research is needed to tease apart the potential pathways (i.e. fertilisation effects or direct consumption) that this detritus enters the food web (e.g. expanding on isotope experiments by [50–52]). Furthermore, the role of detrital subsidies in changing benthic ecosystem function may be enhanced over the larger spatial scales that are characteristic of washed-up detrital matter in temperate intertidal ecosystems (e.g. wrack accumulations, [53]), and this would be worthy of further investigation. Our study, along with previous studies have found that ecosystem responses to detrital addition depend on the detrital source, and this restates that current and projected changes in macrophyte abundance and distributions in temperate estuaries may have implications for connected ecosystems that receive detrital subsidies.

## Supporting Information

S1 Table Sediment properties and ecosystem function data. (XLSX)

S2 Table Macrofaunal community structure data. (A) number of individuals core^-1^ and (B) higher level taxonomic information for each species.
(XLSX)

S3 Table Detrital decomposition and initial carbon and nitrogen content data. (XLSX)

## References

[1] Kelaher BP, Levinton JS. Variation in detrital enrichment causes spatio-temporal variation in soft-sediment assemblages. Mar Ecol Prog Ser. 2003; 261: 85–97. 10.3354/meps261085

[2] Bishop MJ, Kelaher BP. Non-additive, identity-dependent effects of detrital species mixing on soft-sediment communities. Oikos. 2008; 117(4): 531–42. 10.1111/j.2008.0030-1299.16418.x

[3] Olabarria C, Incera M, Garrido J, Rossi F. The effect of wrack composition and diversity on macrofaunal assemblages in intertidal marine sediments. J Exp Mar Bio Ecol. 2010; 396(1): 18–26. 10.1016/j.jembe.2010.10.003

[4] Taylor SL, Bishop MJ, Kelaher BP, Glasby TM. Impacts of detritus from the invasive alga *Caulerpa taxifolia* on a soft sediment community. Mar Ecol Prog Ser. 2010; 420: 73–81. 10.3354/meps08903

[5] Gladstone-Gallagher RV, Lundquist CJ, Pilditch CA. Response of temperate intertidal benthic assemblages to mangrove detrital inputs. J Exp Mar Bio Ecol. 2014; 460: 80–8. 10.1016/j.jembe.2014.06.006

[6] Bishop MJ, Kelaher BP. Impacts of detrital enrichment on estuarine assemblages: disentangling effects of frequency and intensity of disturbance. Mar Ecol Prog Ser. 2007; 341: 25–36. 10.3354/meps341025

[7] Rossi F, Underwood AJ. Small-scale disturbance and increased nutrients as influences on intertidal macrobenthic assemblages: experimental burial of wrack in different intertidal environments. Mar Ecol Prog Ser. 2002; 241: 29–39. 10.3354/meps241029

[8] Rossi F, Gribsholt B, Gazeau F, Di Santo V, Middelburg JJ. Complex effects of ecosystem engineer loss on benthic ecosystem response to detrital macroalgae. PLoS One. 2013; 8(6): e66650 10.1371/journal.pone.0066650 23805256 PMC3689659

[9] Kelaher BP, Bishop MJ, Potts J, Scanes P, Skilbeck G. Detrital diversity influences estuarine ecosystem performance. Glob Change Biol. 2013; 19: 1909–18. 10.1111/gcb.12162 23505131

[10] Blackburn TH, Blackburn ND, Mortimer RJG, Coleman ML, Lovley DR. Rates of microbial processes in sediments. Philos Trans A Math Phys Eng Sci. 1993; 344(1670): 49–58. 10.1098/rsta.1993.0074

[11] García-Robledo E, Corzo A, de Lomas JG, van Bergeijk SA. Biogeochemical effects of macroalgal decomposition on intertidal microbenthos: a microcosm experiment. Mar Ecol Prog Ser. 2008; 356: 139–51. 10.3354/meps07287

[12] Lohrer AM, Hewitt JE, Hailes SF, Thrush SF, Ahrens M, Halliday J. Contamination on sandflats and the decoupling of linked ecological functions. Austral Ecol. 2011; 36(4): 378–88. 10.1111/j.1442-9993.2010.02148.x

[13] García-Robledo E, Revsbech NP, Risgaard-Petersen N, Corzo A. Changes in N cycling induced by *Ulva* detritus enrichment of sediments. Aquat Microb Ecol. 2013; 69(2): 113–22. 10.3354/ame01626

[14] Rodil IF, Lohrer AM, Thrush SF. Sensitivity of heterogeneous marine benthic habitats to subtle stressors. PLoS One. 2013; 8(11): e81646 10.1371/journal.pone.0081646 24312332 PMC3842950

[15] Rossi F. Small-scale burial of macroalgal detritus in marine sediments: Effects of *Ulva* spp. on the spatial distribution of macrofauna assemblages. J Exp Mar Bio Ecol. 2006; 332(1): 84–95. 10.1016/j.jembe.2005.11.003

[16] Bishop MJ, Kelaher BP. Replacement of native seagrass with invasive algal detritus: impacts to estuarine sediment communities. Biol Invasions. 2013; 15(1): 45–59. 10.1007/s10530-012-0267-0

[17] Bishop MJ, Kelaher BP. Context-specific effects of the identity of detrital mixtures on invertebrate communities. Ecol Evol. 2013; 3(11): 3986–99. 10.1002/ece3.775 24198954 PMC3810889

[18] Moore JC, Berlow EL, Coleman DC, de Ruiter PC, Dong Q, Hastings A, et al Detritus, trophic dynamics and biodiversity. Ecol Lett. 2004; 7(7): 584–600. 10.1111/j.1461-0248.2004.00606.x

[19] Hyndes GA, Lavery PS, Doropoulos C. Dual processes for cross-boundary subsidies: incorporation of nutrients from reef-derived kelp into a seagrass ecosystem. Mar Ecol Prog Ser. 2012; 445: 97–107. 10.3354/meps09367

[20] Underwood GJC, Kromkamp JC. Primary production by phytoplankton and microphytobenthos in estuaries. Adv Ecol Res. 1999; 29: 93–153.

[21] Bishop MJ, Coleman MA, Kelaher BP. Cross-habitat impacts of species decline: response of estuarine sediment communities to changing detrital resources. Oecologia. 2010; 163(2): 517–25. 10.1007/s00442-009-1555-y 20063171

[22] Gladstone-Gallagher RV, Lundquist CJ, Pilditch CA. Mangrove (*Avicennia marina* subsp. *australasica*) litter production and decomposition in a temperate estuary. N Z J Mar Freshw Res. 2014; 48(1): 24–37. 10.1080/00288330.2013.827124

[23] Ainley LB, Bishop MJ. Relationships between estuarine modification and leaf litter decomposition vary with latitude. Estuar Coast Shelf Sci. 2015; 164: 244–52. 10.1016/j.ecss.2015.07.027

[24] Albright LJ, Chocair J, Masuda K, Valdes M. *In situ* degradation of the kelps *Macrocycstis integrifolia* and *Nereocystis Luetkeana* in British Columbia coastal waters. Le Naturaliste Canadien. 1980; 107(1): 3–10.

[25] Wieder RK, Lang GE. A critique of the analytical methods used in examining decomposition data obtained from litter bags. Ecology. 1982; 63(6): 1636–42. 10.2307/1940104

[26] Bishop MJ, Kelaher BP, Alquezar R, York PH, Ralph PJ, Skilbeck CG. Trophic cul-de-sac, *Pyrazus ebeninus*, limits trophic transfer through an estuarine detritus-based food web. Oikos. 2007; 116(3): 427–38. 10.1111/j.2006.0030-1299.15557.x

[27] O’Brien AL, Morris L, Keough MJ. Multiple sources of nutrients add to the complexities of predicting marine benthic community responses to enrichment. Mar Freshw Res. 2010; 61(12): 1388–98. 10.1071/MF10085

[28] Woodroffe CD. Litter production and decomposition in the New Zealand mangrove, *Avicennia marina* var. *resinifera*. N Z J Mar Freshw Res. 1982; 16(2): 179–88. 10.1080/00288330.1982.9515961

[29] Turner SJ. Growth and productivity of intertidal *Zostera capricorni* in New Zealand estuaries. N Z J Mar Freshw Res. 2007; 41(1): 77–90.

[30] Singleton P. Draft Whangamata Harbour Plan: Looking forward to a healthier harbour. Report prepared for Environment Waikato (internal report 2007/14), Hamilton New Zealand, 2007; 1–84. Available: http://www.waikatoregion.govt.nz/PageFiles/1207/draftwhangamataharbourplan.pdf

[31] Needham H, Townsend M, Hewitt J, Hailes S. Intertidal habitat mapping for ecosystem goods and services: Waikato estuaries. Report prepared for Waikato Regional Council (Technical report 2013/52), Hamilton, New Zealand, 2013; 1–64. Available: http://www.waikatoregion.govt.nz/PageFiles/27981/TR201352.pdf

[32] Lohrer AM, Halliday NJ, Thrush SF, Hewitt JE, Rodil IF. Ecosystem functioning in a disturbance-recovery context: Contribution of macrofauna to primary production and nutrient release on intertidal sandflats. J Exp Mar Bio Ecol. 2010; 390(1): 6–13. 10.1016/j.jembe.2010.04.035

[33] Arar EJ, Collins GB. Method 445.0: *In vitro* determination of chlorophyll *a* and pheophytin *a* in marine and freshwater algae by fluorescence Cincinnati, Ohio, USA: U.S. Environmental Protection Agency; 1997.

[34] Anderson RO. A modified flotation technique for sorting bottom fauna samples. Limnol Oceanogr. 1959; 4(2): 223–5. 10.4319/lo.1959.4.2.0223

[35] Lohrer AM, Thrush SF, Gibbs MM. Bioturbators enhance ecosystem function through complex biogeochemical interactions. Nature. 2004; 431(7012): 1092–5. 10.1038/nature03042 15470385

[36] Thrush SF, Hewitt JE, Gibbs M, Lundquist C, Norkko A. Functional role of large organisms in intertidal communities: Community effects and ecosystem function. Ecosystems. 2006; 9(6): 1029–40. 10.1007/s10021-005-0068-8

[37] Anderson MJ, Gorley RN, Clarke KR. PERMANOVA A+ for PRIMER: Guide to software and statistical methods UK: PRIMER-E Ltd, Plymouth Marine Laboratory; 2008.

[38] Clarke KR, Gorley RN. PRIMER v6: User manual/tutorial UK: PRIMER-E Ltd, Plymouth Marine Laboratory; 2006.

[39] Hewitt J, Thrush S, Gibbs M, Lohrer D, Norkko A. Indirect effects of *Atrina zelandica* on water column nitrogen and oxygen fluxes: The role of benthic macrofauna and microphytes. J Exp Mar Bio Ecol. 2006; 330(1): 261–73. 10.1016/j.jembe.2005.12.032

[40] Braeckman U, Foshtomi MY, Van Gansbeke D, Meysman F, Soetaert K, Vincx M, et al Variable importance of macrofaunal functional biodiversity for biogeochemical cycling in temperate coastal sediments. Ecosystems. 2014; 17(4): 720–37. 10.1007/s10021-014-9755-7

[41] Pratt DR, Lohrer AM, Pilditch CA, Thrush SF. Changes in ecosystem function across sedimentary gradients in estuaries. Ecosystems. 2014; 17: 182–94. 10.1007/s10021-013-9716-6

[42] Morrisey DJ, Underwood AJ, Howitt L, Stark JS. Temporal variation in soft-sediment benthos. J Exp Mar Bio Ecol. 1992; 164(2): 233–45. 10.1016/0022-0981(92)90177-C

[43] Thrush SF, Pridmore RD, Hewitt JE. Impacts on soft-sediment macrofauna: The effects of spatial variation on temporal trends. Ecol Appl. 1994; 4(1): 31–41. 10.2307/1942112

[44] Thrush SF, Halliday J, Hewitt JE, Lohrer AM. The effects of habitat loss, fragmentation, and community homogenization on resilience in estuaries. Ecol Appl. 2008; 18(1): 12–21. 10.1890/07-0436.1 18372552

[45] Hewitt J, Thrush S, Lohrer A, Townsend M. A latent threat to biodiversity: consequences of small-scale heterogeneity loss. Biodivers Conserv. 2010; 19(5): 1315–23. 10.1007/s10531-009-9763-7

[46] Lohrer AM, Thrush SF, Hewitt JE, Kraan C. The up-scaling of ecosystem functions in a heterogeneous world. Sci Rep. 2015; 5: 10349 10.1038/srep10349 25993477 PMC4438619

[47] Arnold TM, Targett NM. Marine tannins: the importance of a mechanistic framework for predicting ecological roles. J Chem Ecol. 2002; 28(10): 1919–34. 10.1023/a:1020737609151 12474891

[48] Alongi DM. The influence of mangrove-derived tannins on intertidal meiobenthos in tropical estuaries. Oecologia. 1987; 71(4): 537–40. 10.2307/4218197 28312223

[49] Miller DC, Geider RJ, MacIntyre HL. Microphytobenthos: The ecological role of the "secret garden" of unvegetated, shallow-water marine habitats. II. Role in sediment stability and shallow-water food webs. Estuaries. 1996; 19(2): 202–12.

[50] Rossi F. Recycle of buried macroalgal detritus in sediments: use of dual-labelling experiments in the field. Mar Biol. 2007; 150(6): 1073–81. 10.1007/s00227-006-0438-6

[51] Rossi F, Incera M, Callier M, Olabarria C. Effects of detrital non-native and native macroalgae on the nitrogen and carbon cycling in intertidal sediments. Mar Biol. 2011;158(12):2705–15. 10.1007/s00227-011-1768-6

[52] Oakes JM, Connolly RM, Revill AT. Isotope enrichment in mangrove forests separates microphytobenthos and detritus as carbon sources for animals. Limnol Oceanogr. 2010;55(1):393–402. 10.4319/lo.2010.55.1.0393

[53] Rodil IF, Olabarria C, Lastra M, López J. Differential effects of native and invasive algal wrack on macrofaunal assemblages inhabiting exposed sandy beaches. J Exp Mar Bio Ecol. 2008; 358(1): 1–13. 10.1016/j.jembe.2007.12.030

